# “The Walls Had Been Built”: A Qualitative Study of Canadian Adolescent Perspectives on Mental Health During the COVID-19 Pandemic

**DOI:** 10.1177/23333936241273270

**Published:** 2024-09-02

**Authors:** Mischa Taylor, Gina Dimitropoulos, Shannon D. Scott, Shelly Ben-David, Carla Hilario

**Affiliations:** 1 University of British Columbia Okanagan Campus, Kelowna, Canada; 2 University of Calgary, Calgary, Canada; 3 University of Alberta, Edmonton, Canada

**Keywords:** adolescents, mental health, COVID-19, Canada, youth engaged, public health measures

## Abstract

Public health measures (PHMs) implemented during the COVID-19 pandemic introduced sudden changes to adolescents’ everyday routines and required adolescents to repeatedly adapt their routines at a critical developmental stage. While meant to protect physical health, the PHMs destabilized mental health. Using a youth-engaged approach and interpretive description, this study explored adolescents’ perspectives on their mental health in relation to the COVID-19 pandemic-related PHMs in Canada from March 2020 to the time of data collection in March 2022. Four Youth Research Collaborators contributed an adolescent lens to informing study activities, and a total of 33 high-school aged adolescents ages 14 to 19 completed individual interviews. Findings suggest an overarching concept of a “timeline” through which adolescents described their experiences. Most adolescents described their mental health as worsening during the initial lockdown, although some adolescents experienced positive mental health outcomes. Several adolescents felt their mental health had not recovered after the PHMs were fully lifted. This study contributes young Canadians’ unique voices to the literature on the pandemic-related PHMs and adolescent mental health. It is essential that the impacts of the pandemic on adolescent mental health continue to be a focus of research and programming to better understand and address its ongoing effects.

## Introduction

When the global novel coronavirus (COVID-19) pandemic began in 2020, governments worldwide implemented public health measures (PHMs) to control and reduce its spread. In several countries, periods of stringent PHMs corresponded to higher infection rates and states of emergency. In Canada, PHMs included online school, physical distancing, the closure of most public spaces, and canceling of group and social events (Canadian Institute for Health Information [CIHI], 2022). These measures introduced sudden and sweeping changes to everyday routines, particularly for adolescents whose educational, social, familial, and community environments dramatically shifted. During periods known as “waves” more PHMs were in place, school transitioned to online, in-person gatherings with friends were prohibited, and extracurricular activities were canceled. However, measures remained in place to greater or lesser degrees throughout the pandemic with restrictions being implemented, lifted, and reinstituted as infection rates changed. As the PHMs evolved and fluctuated, adolescents were required to continually adapt their lifestyles and routines accordingly, triggering significant uncertainty, anxiety, and stress (Montreuil et al., 2023; Richard et al., 2023). The pandemic and accompanying measures deprived adolescents of social support and adaptive coping strategies, thereby potentially elevating the risk of maladaptive responses and increasing the potential for decreased mental health outcomes. The purpose of this study was to investigate high-school aged adolescents’ perspectives on their mental health in relation to the COVID-19 pandemic-related public health measures.

### Background

Mental health amongst Canadian adolescents has been an issue of growing concern predating the COVID-19 pandemic, as diagnoses of mood and anxiety disorders have continued to increase over time (Wiens et al., 2020), and adolescent suicide rates remain high (Vaillancourt et al., 2021). This trend is evident in other nations as well, as adolescent mental health disorders in Europe were reported to be higher than the global average in 2020 (UNICEF, 2021), and 42% of American adolescents described persistent feelings of sadness or hopelessness in 2021, compared to 28% in 2011 (Centres for Disease Control and Prevention, 2023). At a time of transition from childhood to adulthood when significant psychological development occurs, high-school aged adolescents can be especially vulnerable to mental health challenges (Boak et al., 2018; Brooks et al., 2002). Mental health is also impacted by social determinants of health such as gender, socio-economic status, and ethnicity (Silva et al., 2016; Viner et al., 2012), and mental health outcomes for adolescents often differ according to these factors. At such a critical developmental stage as adolescence when behaviors and mental health conditions often develop (Kessler et al., 2005; World Health Organization, 2012), the length and severity of the pandemic and corresponding PHMs introduced an additional environmental factor for adolescents to navigate, with the potential for lasting mental health impacts (Fegert et al., 2023; Wade et al., 2023). Although meant to protect physical health, the PHMs had the potential to destabilize mental health as well.

Since the start of the pandemic, adolescent mental health has been a major focus of study. Several studies to date have created a snapshot of adolescent mental health during a specific wave or at a key point (i.e., Chen et al., 2020; Hansen et al., 2022; S. E. Jones et al., 2022; Richard et al., 2023), while others have applied a longitudinal lens to compare adolescent mental health prior to and during the pandemic (i.e., Magson et al., 2021; Pedrini et al., 2022; Rosen et al., 2021; Sayal et al., 2023; Wade et al., 2023). Many studies have examined adolescent mental health during the first wave of the pandemic when the virus initially spread and PHMs were most stringent (i.e., Citerne et al., 2023; Ellis et al., 2020; Thomas et al., 2022). Most frequently, studies during this period found that mental health overall worsened as anxiety and depression increased (Ellis et al., 2020; E. A. K. Jones et al., 2021). In other cases, such as studies with adolescents in China and middle-upper class adolescents in France, found that mental health was either unaffected or improved during the first wave as they engaged in physical activity or maintained social connections remotely (Chen et al., 2020; Citerne et al., 2023). Other studies have examined adolescent mental health following the first wave when PHMs fluctuated significantly in the pandemic’s first and second years (i.e., Beames et al., 2021; Bell et al., 2023; Racine et al., 2021; Stewart et al., 2023). There is some evidence that adolescent mental health fluctuated according to the quantity and severity of PHMs in place at the time (Montreuil et al., 2023). By contrast, other studies have reported that adolescent mental health worsened as the pandemic continued into months and years (Bell et al., 2023; Racine et al., 2021; Richard et al., 2023), or as some PHMs were lifted and “normal” routines were in part restored (Hansen et al., 2022; Stewart et al., 2023). For example, as online school mandates were lifted in the United States in fall 2020 and spring 2021, allowing adolescents to return to in-person school, teen suicide rates increased by 2% to 18% (Hansen et al., 2022). A study of high school adolescents in particular found that amongst this age group, more than a third reported poor mental health during the first half of 2021 as a result of the pandemic and PHMs (S. E. Jones et al., 2022). These studies indicate that mental health was affected, albeit in different ways and to varying extents, at different points throughout the pandemic.

Longitudinal studies have investigated the impacts of the pandemic on adolescent mental health by comparing indicators of mental health before and during the pandemic. Compared to prior to the pandemic, several studies found a decline in self-reported mental wellness at a given point in the pandemic (Gustafsson et al., 2023; Magson et al., 2021), or an increase in internalizing disorders (Hawke et al., 2020; Rosen et al., 2021), in part or in whole due to the restrictions imposed by the PHMs. Not all longitudinal studies found, however, that mental health declined with more PHMs. For instance, a study of adolescents with previously diagnosed emotional difficulties found that their mental health was lowest when PHMs were relaxed and in-person schooling returned (Sayal et al., 2023), suggesting that although PHMs did impose the same restrictions on adolescents’ daily routines, the subsequent effects on mental health differed. Notably, the bulk of longitudinal studies have focused on pre- and trans-pandemic comparisons, overlooking any mental health changes once PHMs were removed (Wade et al., 2023).

While there is ample evidence to indicate that mental health amongst adolescents both in Canada and elsewhere was affected at a specific point or in comparison with pre-pandemic mental health, to our knowledge very few studies have explored adolescents’ perceptions of their mental health across all waves of the pandemic, as well as after PHMs were removed. As adolescent mental health remains a pressing concern, understanding how their mental wellness might have shifted after the PHMs were lifted is especially critical to discerning and addressing the residual, long-term mental health effects that may persist. We refer to this period after the PHMs were removed as “post-PHMs.” Data from this study speaks to adolescent mental health in the later waves and post-PHMs as well as the earlier waves, based on their reflections. This article highlights the range of experiences and mental health impacts shared by participants.

### Purpose and Research Questions

The purpose of this study was to investigate high-school aged adolescents’ perspectives on their mental health in relation to the COVID-19 pandemic-related public health measures. While the study was conducted in 2022, the research was intended to elicit and document participant perspectives on their experiences at any time during the pandemic in Canada, from March 2020 to the time of data collection in March 2022. This study explored the following research questions: (1) How did the pandemic-related public health measures shape the everyday lives of adolescents across the pandemic? And; (2) What are the perspectives of adolescents on how the pandemic has affected their mental health?

## Methods

### Study Design and Setting

We used a youth engagement approach (Hawke et al., 2018) and interpretive description (Thorne, 2016) to explore how the COVID-19 pandemic-related public health measures affected adolescents’ everyday lives and mental health. Four adolescents were recruited at the onset of the study to become Youth Research Collaborators (YRCs) and were engaged over an 18-month period. Data collection was conducted remotely to align with COVID-19 protocols in place when the study began, and for consistency later once the physical distancing protocols had been lifted. Interviews were conducted with adolescents from two sites in Alberta, a province located in western Canada: one metropolitan site of about 1.5 million people (Edmonton), and a second site including a small sized city and a town of less than 20,000 people, referred to as “Grove County” in this article. Interviews were conducted in two sites to capture experiences from both smaller and larger population centers. The volume of rich data from this study necessitated two manuscripts, each reporting unique data; the second manuscript related to this study is currently under review, which applies the social determinants of health to investigating the impacts of PHMs on adolescent mental health (Taylor et al., 2024). This study was reviewed and approved by the affiliated universities’ research ethics boards: University of Alberta Research Ethics Board (Pro00113687), University of British Columbia Research Ethics Board (H22-03687), and University of Calgary Research Ethics Board (REB22-0099).

### Youth Research Collaborators

We used a youth engagement approach to foreground adolescents’ experiences and expertise throughout the study (Hawke et al., 2018). Four Youth Research Collaborators (YRCs) were recruited to join the study and contribute an adolescent lens to inform its activities. YRCs were ages 15 and 16 when the study began, and were living in the same region of Alberta as study participants. A diagnosed mental health condition or other specific experiences or qualifications were not required to become a YRC in this study. The project community partner organization, a youth-serving agency based in Alberta, supported YRC recruitment.

Throughout the course of the study, YRCs met with the research team to learn about the research process and inform the design and implementation of study activities. Specifically, YRCs supported the recruitment process by sharing feedback on recruitment materials and assisting with study promotion within their networks; data collection by reviewing and revising the interview guide, including the addition of questions specific to timelines during the COVID period; data analysis by providing feedback on preliminary themes based on how they related to YRCs’ perspectives; and knowledge mobilization by sharing feedback on presentations and project reports (Csillag et al., 2023).

When interpreting the data, the YRCs provided feedback on preliminary findings that was critical to identifying patterns and deciding how to organize the results. Notably, YRCs identified the value of using a timeline as the organizing framework for their experiences. YRCs felt that changing adolescent mental health as the PHMs were implemented or lifted, and the impact of time as they were extended over 2 years, was a foundational and common experience for adolescents. The research team integrated this perspective into the data analysis so that the findings presented in this article reflect adolescents’ interpretations and priorities, and are relevant to the target population (Hawke et al., 2018; Jacquez et al., 2013).

### Sampling and Recruitment

Adolescents aged 14 to 19 years living in either research site during the pandemic were invited to participate in the study. This age range was selected to explore the unique experiences of a particular cohort, namely, older adolescents in the secondary or high school age group who would likely have experienced similar conditions, and changes to these conditions, during the pandemic. We recruited adolescents who self-identified as having experienced mental health challenges as well as those who had received a diagnosis of a mental disorder before or during the pandemic. Adolescents with no experience of mental health challenges prior to or during the pandemic were also invited to participate so that a range of mental health backgrounds were represented.

In alignment with a youth engaged approach, convenience and snowball sampling (Bhardwaj, 2019) were used to recruit participants through the project’s community partner organization, YRCs, and other youth-serving organizations in the two sites. Experiential insight on adolescent recruitment from the community partner organization and YRCs helped to streamline the recruitment strategy to include sharing recruitment materials at junior and senior high schools and through relevant listservs. Participants were also encouraged to share the study information with their peers after their interview. Rigor in participant sampling was upheld by having clearly defined research questions that guided the identification and recruitment of the study sample, using sampling strategies that were appropriate and relevant to using an engaged approach, and using multiple sources to recruit and select participants (Morse et al., 2002).

Adolescents interested in the study contacted the research team and were subsequently given a secure email link to access the study information and provide virtual informed consent using REDCap, a secure web application (Harris et al., 2009). Participants aged 15 to 19 were considered able to understand the study’s purpose, risks, and benefits, and provided consent on their own. One participant was aged 14, and their assent was gathered alongside parental consent. Following their interview, participants were emailed a $25 gift card to acknowledge their time, expertise, and contributions. In total, 33 adolescents aged 14 to 19 years old participated in this study.

### Data Collection

Thirty-three interviews were conducted by the lead author (MT), who is trained in qualitative data collection, between May and October 2022. In developing the interview guide, YRCs were involved in collaboratively reviewing and refining its language, phrasing, and organization to enhance its relevance, resonance, and clarity for study participants. YRCs also identified additional research questions to further elicit adolescents’ experiences. The interview guide was then piloted with three participants and subsequently refined in collaboration with YRCs to improve phrasing and organization where needed. In interviews lasting between 40 and 90 min, participants responded to questions about how the public health measures have shaped their daily lives and routines, and any mental health changes they experienced at different points across the pandemic. Participants reflected on their mental health prior to the pandemic, at various points during the pandemic, and following the formal end of PHMs (March, 2022) in Alberta. Data collection concluded when the data gathered reflected a reasonable range of adolescent experiences to uphold data adequacy and appropriately address the study purpose (Thorne, 2020; Varpio et al., 2017).

### Participant Characteristics

Participants fairly equally represented both study sites, with 16 living in Grove County and 17 living in the urban metropolitan site. More adolescents who identified as young women participated in the study (*n* = 18), as did older adolescents aged 18 or 19 (*n* = 20). Participant characteristics are described in Table 1.

**Table 1.** Participant Demographics.

Demographic categories	*N* (33)	Percentage (100%)
Residence during pandemic		
Rural site	16	48.5
Urban site	17	51.5
Gender identity at time of interview		
Identify as young woman	18	54.5
Identify as young man	12	36.4
Identify as other	2	6.1
Undisclosed	1	3.0
Age at time of interview		
14	1	3.0
15	2	6.1
16	5	15.15
17	5	15.15
18	12	36.4
19	8	24.2

### Data Analysis

Recordings were transcribed verbatim using Zoom Audio Transcript feature (Zoom Support, 2022) and then accuracy checked and de-identified by a research assistant which included assigning pseudonyms. Guided by an interpretive description approach and constant comparative analysis (Boeije, 2002), transcripts were inductively coded to identify and compare patterns of similarity and difference. Initial coding was completed concurrently with interviews so that data collection and analysis could iteratively inform both processes. Using Nvivo 12 software, the lead author (MT) inductively generated initial codes. Following the process of constant comparative analysis (Boeije, 2002), coded excerpts within a single interview were compared to explore consistency and variability of each participant’s experience over time. Excerpts were also compared between interviews to identify similarities and differences across participants’ experiences. Codes were then refined with input from YRCs and other research team members, and finalized with the PI (CH) into a codebook. Next, codes were grouped together into parent codes informed by the process of comparison within and across interviews so that parent codes reflected similarities and differences of participant experiences. Guided by feedback from the YRCs, findings were then mapped onto a timeline of the PHMs in Alberta to facilitate interpretation of the patterns around time, which informed the final organization of the data into time period categories during and following the pandemic. Comparisons of participants’ experiences by age and gender identity were also conducted to identify any patterns within these demographic groups.

During data collection and analysis, research team members completed memos to track insights and observations and kept a study log to record decisions and steps in the analysis process. The use of a study log, prolonged period of data collection, and team approach to data analysis helped to increase trustworthiness and validity of the findings. The following section describes the findings from our study that have been interpreted with discussion from YRCs, who suggested the organizing framework of a “timeline” concept in presenting the results.

## Findings

**Figure 1.**
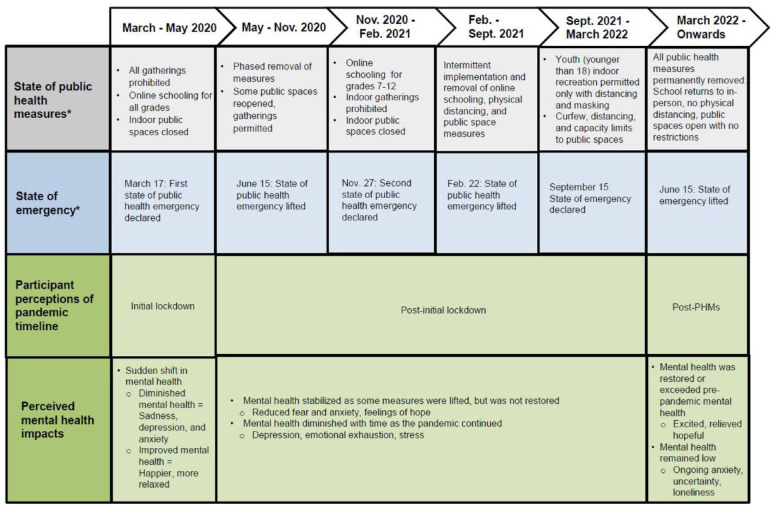
Timeline of Alberta public health measures, states of emergency, and participants’ perceptions of the pandemic timeline and mental health impacts (CIHI, 2022).

A prominent pattern across the perspectives of high-school aged adolescents in this study relates to the notion of time during the various waves of the COVID-19 pandemic. Participants described their mental health according to a “timeline,” or different time periods during the pandemic. In the research context of Alberta, public health measures were in place in greater or lesser numbers and severity between March 2020 and March 2022. Although three states of emergency formally demarcated changing infection rates and PHMs into “waves,” participants generally divided the years of the pandemic according to the following categories: Initial lockdown (March–May 2020); Post-initial lockdown (June 2020–March 2022); and Post-PHMs (March 2022 onwards). The initial lockdown period was characterized by public health measures in full effect, including online school for all students, the closing of all indoor public spaces, and no indoor gatherings of any size outside of households. The initial lockdown period ended in May 2020, and from then until March 2022, there were several fluctuations in the extent and severity of the PHMs as states of emergency came into effect or were lifted. This “in-between” period, where PHMs or the possibility of PHMs were still present but to a lesser extent than the initial lockdown, was generally considered by participants as a single time period as their lives remained in a state of flux throughout various changes to PHMs. In March 2022, all PHMs were fully lifted in the province of Alberta, and participants viewed the “post-PHMs” as a third distinct time period. The timeline shown in Figure 1 maps these time periods onto the formal timeline of PHMs in the province over the full 2 years of restrictions, and summarizes the main impacts on mental health throughout each time period, as described in more detail in the time period categories below.

In the months directly preceding the pandemic’s onset, the majority of participants felt that their overall mental health was “good.” Participants gave various reasons for this such as having nurturing relationships, extracurricular activities, and positive educational experiences prior to the pandemic. Although comparisons of participants’ experiences by gender identity were conducted, no differences were found. The findings presented here describe participants’ perspectives on the PHMs’ effect on their daily lives and mental health across the course of the whole pandemic and after the measures were formally removed. Pseudonyms are used throughout the findings section when referencing participants. Findings are organized according to the time periods identified by participants: Initial lockdown, Post-initial lockdown, and Post-PHMs.

### Initial Lockdown: “A Hold on Life”

The first 3 months (March–May 2020) of the pandemic were described by adolescents as having the most stringent public health measures compared to the following months. Public health measures in the first months limited nearly all aspects of daily life that involved interaction with others, including the closing of non-essential services and public spaces; canceling of events and activities; and transitioning school to online, remote delivery. Many participants discussed the initial lockdown as distinct compared to later in the pandemic. Participants described their lives as being affected in varying ways, with implications for their mental health. This section describes: Impact of initial lockdown on daily life and routine; Emotional response during the initial lockdown; and Mental health implications.

### Impact of Initial Lockdown on Daily Life and Routine

The majority of participants had been involved in sports teams, clubs, and community activities that took place in person prior to the pandemic, and so the implementation of PHMs during the initial lockdown created a sudden and substantial shift to their everyday lives. As the public health measures came into effect, many aspects of their lives changed: “[I] couldn’t see my friends, no school, no sports, no being outside. . . So it was a lot to go in one piece” (Amy, age 15, Grove County). Younger participants expressed being particularly impacted by changes to schooling and the subsequent effects on their social connections, however, the impacts were both extensive and challenging for all adolescents, as “literally everything changed” (Peter, age 18, Edmonton). For instance, according to one adolescent,

When the pandemic started everything turned upside down for me, because the freedom I had before the pandemic was no longer there, the social relationships I had were not there. My physical wellbeing too was unstable, and so I would say that when the pandemic started everything just turned opposite for me, in a negative way. (Stacey, age 18, Edmonton)

The transition to more time spent at home was particularly difficult, leaving participants feeling “trapped” (Trisha, age 17, Grove County) and “lost” (Nicholas, age 19, Edmonton). The extended change to routine created the perception for some adolescents that they had missed key life experiences:

We took a break [for the pandemic] in grade 10 and. . . now I’m graduating and it blows my mind cuz it feels like I never got that time to really have the teenage or high school experience, like everyone else. (Amanda, age 18, Grove County)

Older participants wondered about the impact of the public health measures on their future plans and opportunities: “You feel like now you are about to go out in the world, you are about to go to college. . . And then this thing comes. So what now?” (Reggie, age 18, Edmonton).

Changing routines were easier for some adolescents to adapt to than others. Several participants appreciated fewer academic expectations in particular. As a busy, engaged student, Lisa (age 15, Edmonton) perceived the lockdown “as a time to relax” given that in-class instruction time was much shorter via online schooling. For another participant, “since I had a lot of activities [pre-Covid] it felt really nice to finally have a break. . . It felt like I was on a five-month vacation” (Trisha, age 17, Grove County). By focusing on the life-saving purpose the measures were serving, it was also easier to accept the PHMs: “The measures that were put in, I understood. My daily routine was disrupted yes, but that was the least of my worries. I was more concerned with being alive” (Nicholas, age 19, Edmonton). Although routines were affected for all adolescents, its impact varied.

### Emotional Response During the Initial Lockdown

A range of reactions accompanied the implementation of the PHMs. As participants tried to make sense of the measures and simultaneously adjust to the changes they imposed on their routines, participants expressed feeling “shocked” (Amy, age 15, Grove County), “restricted” (Christine, age 18, Grove County), and “empty” (Amanda, age 18, Grove County). These feelings contributed to negative emotional responses for several adolescents during the initial lockdown, particularly feelings of anger and frustration. One adolescent shared, “I felt very angry because I felt like I was being denied a chance to interact with my friends, do what I want, travel” (Jacob, age 19, Edmonton). For another participant, the pervasiveness of the public health measures across multiple aspects of her life made her feel both controlled and disenfranchised: “I felt powerless- I couldn’t change it, and that really bothers me” (Amy, age 15, Grove County). Numerous participants spoke about their frustration at the public health measures being directed outwards toward others, including those responsible for implementing the public health measures. For Reggie,

At some point you could be angry with everybody, maybe the government—this thing could have been avoided, maybe they could have placed these measures they are placing now, before. There was just anger and there has to be someone to direct anger at. (Reggie, age 18, Edmonton)

Being young at the time of the pandemic also made it harder to make sense of the PHMs. Reflecting on feeling frustrated and confused as to why she couldn’t spend time with her friends during the pandemic, one adolescent shared “I didn’t really grasp that concept [of the pandemic’s severity], I never fully understood it until I got older. . . I was young and I didn’t understand why [my parents] were doing what they were doing” (Ava, age 16, Grove County). These negative emotions were common amongst adolescents, with mental health consequences.

### Mental Health Implications

The PHMs triggered a shift in mental health amongst nearly all participants. Most adolescents felt that their mental health was at its worst during the pandemic’s initial lockdown when there were the most public health measures in place. The sudden and mandated changes to lifestyle and daily routines were challenging, as “the whole scenario, the whole lockdown, no socializing, the anxiety—all that led to the decrease of the mental health strength” (Donald, age 18, Grove County). Steven, for instance, was separated from his father, who was quarantined at his out-of-town place of work for an extended period. Combined with difficulties adjusting to online school and church activities being canceled, he felt that “it was like our life was ending gradually” (Steven, age 19, Edmonton), and as a result, “it was a very terrible, terrible period” (Steven, age 19, Edmonton).

More than half of participants expressed feeling sad or depressed. According to Sarah, “in that period I almost lost my mind. As an extrovert it was completely new being forced to be indoors. So it affected a lot. I became more depressed, I started keeping more to myself” (Sarah, age 18, Edmonton). Worry or anxiety often accompanied sadness and depression as participants attempted to understand the impact of the lockdown on their daily lives. Participants were especially concerned about how long they could expect disruptions to their everyday lives:

I was worried. When is it going to end? How is it going to end? And how bad is it going to get if we are starting with the lockdown? Are we supposed to be in the lockdown for so long? What is going to happen in the future? I was so worried. (Christine, age 18, Grove County)

Some participants felt that the public health measures triggered a downward spiral as they struggled to adjust and adapt. For instance, according to Kyle (age 18, Grove County), “when you don’t have [social connections and extracurricular activities] your mental health goes down because don’t have that outlet, and then you start finding that outlet in other places, which leads to lower mental health.”

Other adolescents, however, did not find these adjustments as difficult, citing fewer academic expectations and pressures, and more free time and flexibility in their schedules. Some participants felt happier and more relaxed with less academic pressure: “At first I thought it was a good thing, because I knew my marks didn’t go down in school, so I wasn’t really stressed about school anymore” (Cory, age 16, Grove County). One adolescent enjoyed the absence of social demands and time for self-reflection that the public health measures precipitated, and felt her mental health improved compared to before the pandemic (Nicole, age 16, Grove County). Pre-existing mental health conditions also improved in some cases. One participant who had previously experienced social anxiety explained that the “[panic attacks] subsided now, because we’re not going out in public places” (Laura, age 18, Grove County). Although the public health measures shaped daily lives and routines in similar ways, mental health impacts varied according to participants’ perceptions and preferences.

### Post-Lockdown: “Nothing Permanent”

As some aspects of pre-pandemic routines were restored, most participants reported that their mental health improved in the period after the initial lockdown in Canada (June 2020–March 2022). Amy, for example, shared that “going back to school really did help. Even if there were all the [public health] rules at school, it definitely helped to be back” (Amy, age 15, Grove County). Others felt their mental health improved as the pandemic progressed because they now knew what to expect of the PHMs, or that the measures became less onerous later in the pandemic compared to first months. One adolescent explained, “I started developing new ideas on how to adapt to the system, and it became automatic. . . So I slowly became accustomed to the system, and slowly the anxiety started reducing, the fear reducing” (Peter, age 18, Edmonton). Some public health measures were gradually eased. Participants referred to this period, where some PHMs were still in place but others had been lifted, as distinct from the period of the initial lockdown. Some aspects of daily life such as the return to in-person school intermittently resumed, while others, such as most extracurricular activities and large social gatherings, remained on hold.

### Post-Lockdown Mental Health

Participants generally described their mental health in this period as “better” than the initial lockdown but still “poorer” than their pre-pandemic mental wellness. Amy shared that “after the first year [my mental health] started to stabilize a bit more. It was definitely lower [than pre-pandemic], but it was a lot better than it was” (Amy, age 15, Grove County). As a result, “life after the lockdown, it was better than life during the lockdown. . . We were comfortable with it, but we weren’t all that comfortable. But, it was better than the lockdown life” (Steven, age 19, Edmonton). The easing of PHMs also suggested that a post-PHMs future could be possible: “There was hope. At least, we could see it was not the end of everything. Maybe at some point there could be a solution” (Reggie, age 18, Edmonton).

Yet, as the remaining PHMs continued to curtail routines and social interactions to some extent, other participants noticed that their mental health did not improve. For these adolescents, negative emotions worsened as they struggled to cope and adjust in the pandemic’s later months. For instance, according to Kyle, the changes precipitated by the public health measures were “really nice at first, but then over time, not being able to hang out with friends and stuff like that really started to affect me” (Kyle, age 18, Grove County). Another adolescent had initially enjoyed the lockdown period, but described her mental health in the subsequent months this way:

I slowly became grayer and grayer, if you were to look at it that way. If I were to put it in the way of a metaphor, before I was very much like a rainbow and then I slowly became duller and duller and duller. (Chelsey, age 16, Grove County)

When school returned to in-person delivery, some schools opted for two 3-hour classes per day for the remainder of the school year, to make up for time lost during the lockdown period. These extended classes presented an additional challenge for adolescents to navigate. In addition, after months without peers, the social interaction that accompanied in-person school was cumbersome and taxing for some adolescents. This was true for Lisa, whose mental health had improved during the initial lockdown due to fewer social pressures, particularly “all the social interactions that you’re doing at school and all the people you have to talk to” (Lisa, age 15, Edmonton). For Lisa then, the shift back to in-person schooling presented new challenges: “When we were in person. . . it took everything out of me, I found it so emotionally exhausting. . . I got so stressed. . . So that definitely affected [my mental health] . . . That was the hardest thing, readjusting” (Lisa, age 15, Edmonton). As a result, the post-initial lockdown was substantially more challenging for these adolescents compared to the lockdown.

Several adolescents found that their mental health fluctuated according to the public health measures, improving or becoming worse when some measures were lifted and worsening or improving when they were once again implemented. As one adolescent commented,

The first time the PHMs were turned down a bit, I was happy. But as time continued and they were back again, it really made me realize that there is nothing permanent. I was thinking that the pandemic is gone, so we are back to our normal life. So, my mental health [improved] in the first instance. But later on, I think it was constant [low]. (Emily, age 17, Edmonton)

As PHMs were instituted and then lifted, adolescent mental health was affected each time their routines were modified. Laura explained that when public health measures were reinstituted later in the pandemic, her mental health “went a little bit down, because now what we were used to, we had to change our lifestyle again” (Laura, age 18, Grove County). One adolescent, however, felt that her mental health was unchanged by the state of the PHMs throughout the pandemic: “[my mental health] was low the whole time, because even when they reduced the public measures. . . I didn’t really meet up with my friends and colleagues. It was more like the same routine for me, like nothing changed” (Sarah, age 18, Edmonton). For this participant, the post-lockdown period did not necessarily afford more opportunities to socialize; from her perspective, her mental wellness did not improve.

### Post-PHMs Mental Health: “A New Normal”

The last public health measures were lifted in March 2022 in Alberta, initiating the shift to “post-PHMs” where aspects of daily life and routines that were considered normal before the pandemic could be restored. Consequently, adolescents’ routines underwent another shift, as did their mental health. Participants shared how the removal of PHMs impacted their post-PHMs routines, and how their mental health was subsequently affected.

Although conditions for “normal life” had been restored, adolescents’ routines did not automatically or immediately mirror this return to “normal.” After 2 years of public health measures, some adolescents felt that a “new normal’” had been established during the pandemic, which continued after the public health measures were lifted. Some adolescents found that they now preferred the pastimes and routines they had become accustomed to during the pandemic, which subsequently affected social connections with peers: “The excitement of doing things like playing soccer now is not as it was before the pandemic, because during the pandemic we developed different interests. . . Now, we end up allocating [only] a little time to playing [soccer]” (Joshua, age 19, Edmonton).

Reflecting on post-PHMs habits and routines, one adolescent commented, “I feel like after the pandemic, a lot of things changed because now we're all glued to our phones, and I think that the pandemic might have caused that” (Eric, age 14, Edmonton). Another participant’s experience reflected this observation of increased screen time and social media use. Whereas prior to the pandemic Cheryl (age 19, Edmonton) had been very involved in a dance group, the interest she developed during the pandemic of spending time on social media extended past the end of the PHMs. Once the measures were lifted, this adolescent chose not to re-join her dance group and instead continued to spend much of her free time on social media.

Many adolescents described having poorer mental health due to the public health measures. When the measures were lifted, they viewed subsequent improvement in their mental wellness. The ability to resume pre-COVID routines was restorative for the majority of participants, because “now I could start my normal life, interacting with friends after a very long period of not seeing each other. So, it improved my mental health” (Jacob, age 19, Edmonton). With the lifting of PHMs, adolescents’ stress and anxiety lessened: “I felt relieved, the kind of feeling that you get when something that has been pressing on you for a long time gets lifted off you. I could just do what I couldn’t do during Covid” (Nicholas, age 19, Edmonton). Improved mental health was also attributed to renewed hope: “During Covid it was really hard to. . . see a way forward. But now there is hope. . . Now, I got to see that things were getting back to normal and maybe we could still have hope for the future” (Christine, age 18, Grove County).

The extent to which mental health improved differed according to individual experiences. Some participants felt that their mental health improved to a higher level than prior to the pandemic’s start because, during the pandemic, “I could not imagine that the situation would come back to normal. But now, . . . I can say that I was very overexcited. That is maybe why [my mental health] reached higher than before” (Jacob, age 19, Edmonton). Other adolescents felt that their mental health recovered to approximately the same as it had been before the pandemic.

Nearly half of participants, however, described their mental health as worse in the post-PHMs period as they had experienced changes that could not be immediately ameliorated. For Ava, when the patterns and norms that they had adjusted to during the pandemic suddenly shifted again, it was disorienting and challenging:

That level of isolation that went on for that long, even when we were allowed to be connected again, those walls had already been built and we were still in the process of taking them down. So it wasn’t this quick and easy shift back into how things are supposed to be. So, I felt like there was always going to be a little bit of leftover isolation. . . I was one of those people who still had to adjust to connecting with people again because. . . Now all of a sudden, you can talk to people [in person] again. (Ava, age 16, Grove County)

Older participants expressed that the experience of the pandemic shook their sense of security in the future, and so feelings of anxiety and uncertainty outlasted the pandemic itself: “Before Covid, we thought you had your life planned out. Who knew that it was going to affect the whole world, right? And then suddenly there was Covid, and right now you can only hope for the best” (Christine, age 18, Grove County).

The pandemic exacerbated pre-existing mental health conditions in some cases. One participant who had experienced a degree of social anxiety prior to the pandemic found that it was worse in the months following the removal of PHMs as she struggled to re-adjust to large groups of people and public spaces:

It’s the aftermath, I’m noticing it now how my anxiety goes up. . . A lot of social stuff is back and I can’t handle it. [Prior to the pandemic], I wouldn’t feel as completely vulnerable to [being around people]. And I feel like now when it gets really loud, I can’t do anything. I feel really incapacitated in a way that I didn’t before and that’s the hardest thing. (Lisa, age 15, Edmonton)

Despite the multitude of challenges the pandemic introduced, it also inspired self-awareness and personal growth. One participant, for instance, became more invested in mental wellness as a result of the pandemic: “Over Covid [is] when I became passionate about a lot of things and mental health was one of them. . . it took a lot of research for me to understand [that] this is a big deal and people need help” (Jamie, age 16, Grove County). For Amanda, although the pandemic had been very challenging, she also felt the learning opportunities it had afforded had been positive:

As bad as Covid was, I think there definitely were beneficial things that came out of it. Of course I would never want to go back to how Covid was, I’d never want to go back in time and be where I was. But I’m glad I learned what I’ve learned from it. (Amanda, age 18, Grove County)

## Discussion

This study contributes young Canadians’ perspectives and their unique voices to the literature on the COVID-19 pandemic and youth mental health, based on a youth-engaged research study. The findings suggest that mental health amongst high-school aged adolescents in Canada was affected by the PHMs and fluctuated across the extent of the pandemic. Changes to mental health were not homogenous, however. While the majority of participants felt that their mental health worsened, others shared positive mental health experiences. Notably, for many adolescents their mental health continued to suffer after the PHMs were lifted.

Participants’ mental health changed at various points in the pandemic. For instance, several adolescents expressed that their mental health took a sudden and significant downturn during the initial lockdown as they were forced to adjust to the immediate changes imposed on many aspects of their daily lives and routines. When the pandemic extended into months and years, the initial emotions of anxiety and anger transformed into a “constant low” for some adolescents where their mental health was consistently negatively affected. One participant reflected that the ongoing environment of the pandemic contributed to her diminished mental health, like “a rainbow [becoming] duller and duller.” These findings are consistent with other studies that found that the impact of the PHMs persisted past the initial lockdown and shifted with time (Montreuil et al., 2023; Stewart et al., 2023). For instance, Canadian adolescents in a qualitative study expressed that although the initial lockdown was the most distressing, their anxiety and uncertainty transformed into depression as the pandemic continued (Montreuil et al., 2023). These studies suggest that both adapting to the PHMs initially as well as their sustained impact on adolescents’ routines over time may have impacted their mental health.

The mental health impacts of the Covid-19 pandemic have been described as constituting a new type of traumatic stress, characterized by depression, anxiety, and PTSD (Kira et al., 2023). These mental health concerns have been linked to isolated stressors throughout the pandemic such as disruption of routines and isolation, as well as the constant, prolonged exposure to the environment of the pandemic and its associated stressors overall (Kira et al., 2023). Adolescents in our study shared similar experiences and reflections. The complex interaction of events and circumstances that they were exposed to during the pandemic raises concerns about traumatic stress that they may have experienced during the pandemic, which may possibly continue even after PHMs were lifted. For example, our data were collected in the 6 months immediately after PHMs had been fully reversed in Alberta, and at the time of their interview, nearly half of adolescents shared that their mental health was still lower than it had been prior to the pandemic.

These findings align with predictions and growing concerns that the circumstances of the pandemic are likely to precipitate longer-term mental health outcomes that extend past the formal end of the pandemic itself (Fegert et al., 2023; Kathirvel, 2020; Tandon, 2020). A “tsunami of psychiatric illness” was predicted to follow the pandemic (Tandon, 2020, p. 1), and adolescent experiences in this study underscore these concerns. Although there is ample evidence to indicate that adolescent mental health was impacted at a specific point or in comparison with pre-pandemic mental health (i.e., Gustafsson et al., 2023; E. A. K. Jones et al., 2021; Madigan et al., 2023; Magson et al., 2021), the same comparison has not been conducted in the period since PHMs were lifted. Evidence of the effects of the PHMs on adolescent mental health is consequently limited to the time period of the pandemic, leaving unanswered questions around the longer-term impacts and overall broader picture of adolescent mental health in the post-PHMs era. Despite predictions of significantly poorer mental health, there remains a paucity of research focusing on mental health recovery in the post-PHMs period. To our knowledge, this is the first qualitative study of adolescent perspectives that includes the post-PHMs period. As a growing concern before COVID-19, particularly amongst the global North, (Fegert et al., 2023; Wiens et al., 2020), findings from this study raise alarms about the compounding effect of the pandemic on overall mental wellness amongst adolescents, even in the absence of PHMs and the larger pandemic context.

While the PHMs were imposed equally on all adolescents, not everyone was affected in the same way nor experienced the same mental health outcomes. Most participants in this study expressed that their mental health worsened at a specific point or progressively across the pandemic, yet some adolescents responded positively to the PHMs and noticed mental health improvements when more measures were in place. In the post-initial lockdown period when in-person school and select activities resumed, these same participants experienced a dramatic shift in their mental health. Adolescents who had struggled with the PHMs expressed feeling hope, whereas the reintroduction of in-person social interaction after an extended period of social isolation was emotionally exhausting and a primary source of stress for those who had reported improved mental health during periods with more PHMs. The majority of evidence indicates that adolescent mental health was negatively impacted by the pandemic; however, some studies report cases where it remained stable or even improved (i.e., Beames et al., 2021; Chen et al., 2020; Citerne et al., 2023; Pedrini et al., 2022). While there may be any number of reasons for this, Pedrini et al. (2022) suggest that for adolescents who prefer time alone, the PHMs created a protective condition that normalized and even promoted social avoidance. Based on participants’ reflections in the current study, this may help to explain the variation in mental health outcomes that they reported.

Understanding the full spectrum of experiences contributes to understanding how and why adolescent mental health is affected in extreme and enduring events such as COVID-19. Impacts of exposure to the same event or condition are highly contextual, and can catalyze any number of mental health outcomes. Several studies identify key social determinants of health such as gender identity, socio-economic status, and age that may have contributed to better or worse adolescent mental health outcomes during the pandemic. For example, young women and adolescents from low-income families were more likely to experience poor mental health during the pandemic compared to young men and adolescents from families with higher incomes (Beames et al., 2021; Stewart et al., 2023; Thomas et al., 2022). Although outcomes vary, some degree of diminished mental health is frequently reported across the majority of studies of adolescent mental health. This study provides qualitative evidence of lower adolescent mental health during the pandemic while also describing instances where mental health improved or stabilized. Although this study did not identify any differences in experience based on gender identity, older participants, those aged 18 or 19, tended to express more concerns about their future compared to younger participants aged 14 to 17, who more frequently discussed the impacts of the PHMs on their schooling and subsequent relationships. There is a need to further explore cases where adolescent mental health was not affected or positively affected alongside adolescents’ personal characteristics and environmental conditions, to deepen understanding of the contextual factors that support better outcomes.

### Implications and Future Directions

This study indicates that the impacts of the pandemic and PHMs on mental health amongst high-school aged adolescents were dynamic and continued to have negative effects even after the removal of PHMs. As a possible new type of traumatic stress that adolescents may still be experiencing even in the post-PHMs period, it is essential for adolescent mental health stakeholders to keep the COVID-19 context in mind. Service providers, for example, can support adolescents to explore any lingering psychological impacts of the pandemic through targeted programs or individual therapy. Service providers can also play a role in developing strategies to remediate these impacts on adolescents’ mental health and subsequently, their daily lives. Studies have indicated that formal adolescent mental health interventions were an effective means of mental health support during the pandemic (Koper et al., 2022; Van Doorn et al., 2021), and so providing services tailored to promoting psychological recovery and wellness in the post-PHMs period are even more salient.

For researchers, the implications arising from the pandemic need to remain a focus of study, prioritizing investigation of the long-term impacts and identifying pathways to mental health recovery. Rather than viewing the pandemic as an event of the past, its effect on mental health must be part of the current research agenda if present and future mental health supports are to effectively address adolescents’ needs and circumstances (Wade et al., 2023). Echoing the call for greater preventive action in the post-pandemic period by Fegert et al. (2023), there is an impetus for examining the layered and long-term impacts of the pandemic, and continuing to view adolescent mental health through a pandemic lens. According to UNICEF’s report card for children and adolescents in the world’s 38 richest nations, in 2020, several prominent countries within the global North such as the United Kingdom, United States, and Canada, as well as Australia and New Zealand, ranked within the lowest third for child and adolescent mental health (UNICEF Innocenti, 2020). Compounded by negative impacts of the pandemic, the need for coordinated, national-level responses are becoming increasingly clear as well as urgent. Within Canada, as well as elsewhere where a federal approach to adolescent mental health research and practice is absent, an adolescent mental health strategy is an unequivocal step to guide a national response (Vaillancourt et al., 2021). Continuing to prioritize research on the longer-term impacts on adolescent mental health as well as mental health recovery is also critical to establishing an evidence base that can ensure mental health is better attended to in future global health crises (Wade et al., 2023).

The range of perspectives adolescents shared suggests that although general commonalities such as a trend toward diminished mental health over the course of the pandemic were evident, adolescents’ experiences of the PHMS were not homogenous. Identifying and taking into account the spectrum of perceptions and responses, including anger and anxiety as well as relief and happiness may help to produce a more complete picture of adolescent mental health in such unique contexts as the COVID-19 pandemic, and a more nuanced understanding of adolescent mental health overall. The pandemic and PHMs may provide a case study, then, offering insights of why adolescents might be affected differently by the same events, and how their mental health fluctuates in unique circumstances and contexts. Further research is needed to continue examining the range of experiences for adolescents, and how this can be applied to current understandings of mental health for this age group.

Applying adolescents’ experiences and insights to shaping the research process enhances the applicability of results to adolescent mental health service delivery and policy development. The use of YRCs in this study foregrounded adolescent perspectives. Similar studies could be conducted in other provinces or countries, or with specific demographic groups such as adolescents with pre-existing mental health conditions, to explore the extent to which adolescents in other locations or with different backgrounds are similarly affected. In these studies, engaging some adolescents as YRCs can similarly enhance its value and impact for both adolescents and adolescent-serving supports.

### Limitations

Data collection for this study was completed in spring and summer 2022, after PHMs were lifted in Alberta. Consequently, participants’ perspectives of continuing mental health impacts in the post-PHMs period are limited to this timeframe. It would be helpful for future studies to document adolescent perspectives on their mental health and recovery after the passing of more time.

In terms of sample, the majority of participants were recruited through select schools or youth-serving organizations at each site. It is possible, then, that findings of this study are reflective of a certain subgroup of adolescents. The use of snowball sampling may also have contributed to the majority of participants being aged 18 or 19 years old, and their experiences may be overrepresented in this study. Since interviews were conducted remotely via the internet, participation was contingent on having online access, which may have precluded some adolescents’ involvement. Other adolescents may not have felt comfortable participating in an online interview for a variety of reasons, such as lack of privacy at home. Future studies could conduct both remote and in-person interviews to accommodate a broader sample of adolescents. Adolescents who chose to participate in this study are also likely help-seeking individuals, and so this study may overrepresent their experiences compared to those who did not seek out this study or wish to participate. Participant racial and ethnic demographic information were also not collected, and so we are unable to interpret the results accordingly. Further research could collect these demographic details to add additional context to the findings.

## Conclusion

This study presents high-school aged adolescents’ experiences throughout the course of the COVID-19 pandemic, highlighting their perceptions of the impact of the PHMs on their mental health during and post-PHMs. Participants generally divided the pandemic into three time periods: Initial lockdown, post-lockdown, and post-PHMs, and used these as anchor points in describing their experiences related to mental health. While the majority of participants felt that their mental health was negatively affected, the PHMs created conditions that were better suited to some, who felt that the measures benefited their mental health. To our knowledge, this is the first study of adolescent perspectives that includes the post-PHMs period. Notably, findings from this study indicate that mental health challenges experienced during the pandemic may be continuing to impact adolescent mental health, even after PHMs have been lifted. It is critically important, then, to continue examining the impact of the COVID-19 pandemic and subsequent PHMs on adolescent mental health and incorporate this into services and supports. Even though the pandemic itself is behind us, its effect remains, and must continue to be considered in future adolescent mental health research and service provision in the present and going forward.
